# Supplementary material for: IFITM3 restricts virus-induced inflammatory cytokine production by limiting Nogo-B mediated TLR responses
Source: Nat Commun. 2022 Sep 8;13:5294. doi: 10.1038/s41467-022-32587-4 (PMC9454482; doi:10.1038/s41467-022-32587-4)
Supplement: Supplementary file 1 — Supplementary Information [file 41467_2022_32587_MOESM1_ESM.pdf]

**Title: IFITM3 restricts virus-induced inflammatory cytokine production by limiting Nogo-B mediated TLR responses**

**Authors:** Clement M.[1#], Forbester J.L.[1,2#], Marsden, M.[1], Sabberwal, P.[1], Sommerville M.S.[1], Wellington D.[2,3], Dimonte S.[1], Clare S.[4], Harcourt K.[4], Yin Z.[2,3], Nobre L.[5], Antrobus R.[5], Jin, B.[6], Chen, M.[7], Makvandi-Nejad, S.[2], Lindborg J.A[8], Strittmatter, S.M.[8], Weekes, M.P.[5], Stanton R.J.[1], Dong T.[2,3], Humphreys I.R.[1]*

**Affiliations:**

1. Division of Infection and Immunity/Systems Immunity University Research Institute, Cardiff University
2. MRC Human Immunology Unit, MRC Weatherall Institute of Molecular Medicine, Radcliffe Department of Medicine, Oxford University, Oxford OX3 9DS, UK
3. Chinese Academy of Medical Sciences (CAMS) Oxford Institute (COI), University of Oxford, Oxford, UK
4. Wellcome Sanger Institute, Wellcome Genome Campus, Hinxton, Cambridge, CB10 1SA, UK
5. Cambridge Institute for Medical Research, University of Cambridge, Hills Road, Cambridge CB2 0XY, UK
6. Fourth Military Medical University, Xian, China
7. Department of Microbial Pathogenesis, Yale University School of Medicine, New Haven, CT 06536, USA.
8. Departments of Neurology and Neuroscience, Yale University School of Medicine, New Haven, Connecticut 06520

# These authors contributed equally, * corresponding author Ian Humphreys (humpreysir@cardiff.ac.uk).

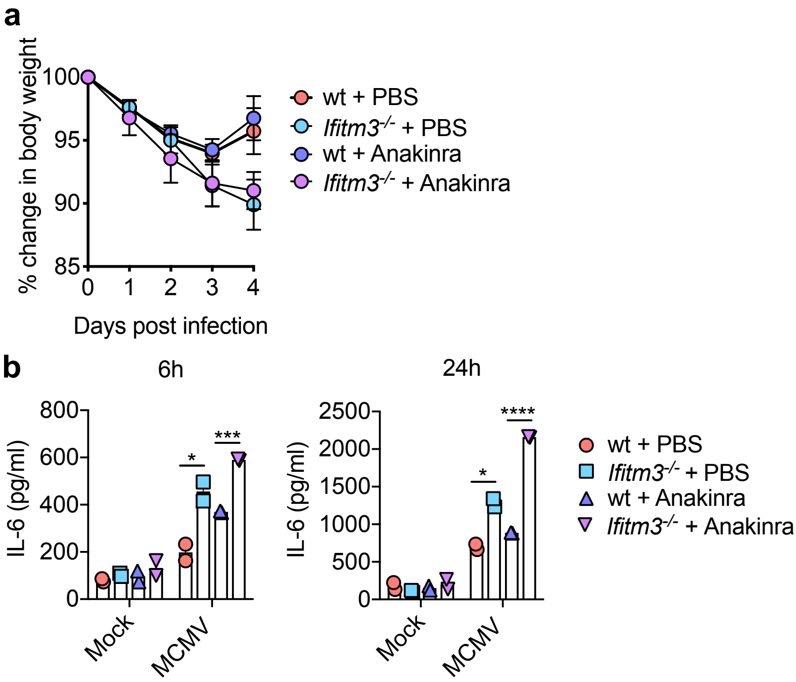


**Supplementary Figure 1**. **Antagonising IL-1R signalling has no impact on MCMV-induced weight loss or cytokine production.** (**a**) Wt and *Ifitm3*$^{-/-}$ mice were infected with MCMV with either 25mg/kg Anakinra or PBS and weight loss was assessed over time. Data shown represents 4 mice per group. (**b)** BM-DCs from wt and *Ifitm3*$^{-/-}$ mice were pre-incubated with or without Anakinra and then infected with MCMV (MOI 1). Supernatant was harvested at 6h and 24 post infection and IL-6 levels were assayed. Data are presented as mean +/- SEM of 2 biologically separate replicates and statistical significance was assessed using 2-way ANOVA (**a**) or 1-way ANOVA (**b**). *P* values are reported as follows: n.s., >0.05; *, ≤0.05; **, ≤0.01; ***, ≤0.001; and ****, ≤0.0001. Source data are provided as a Source Data file.

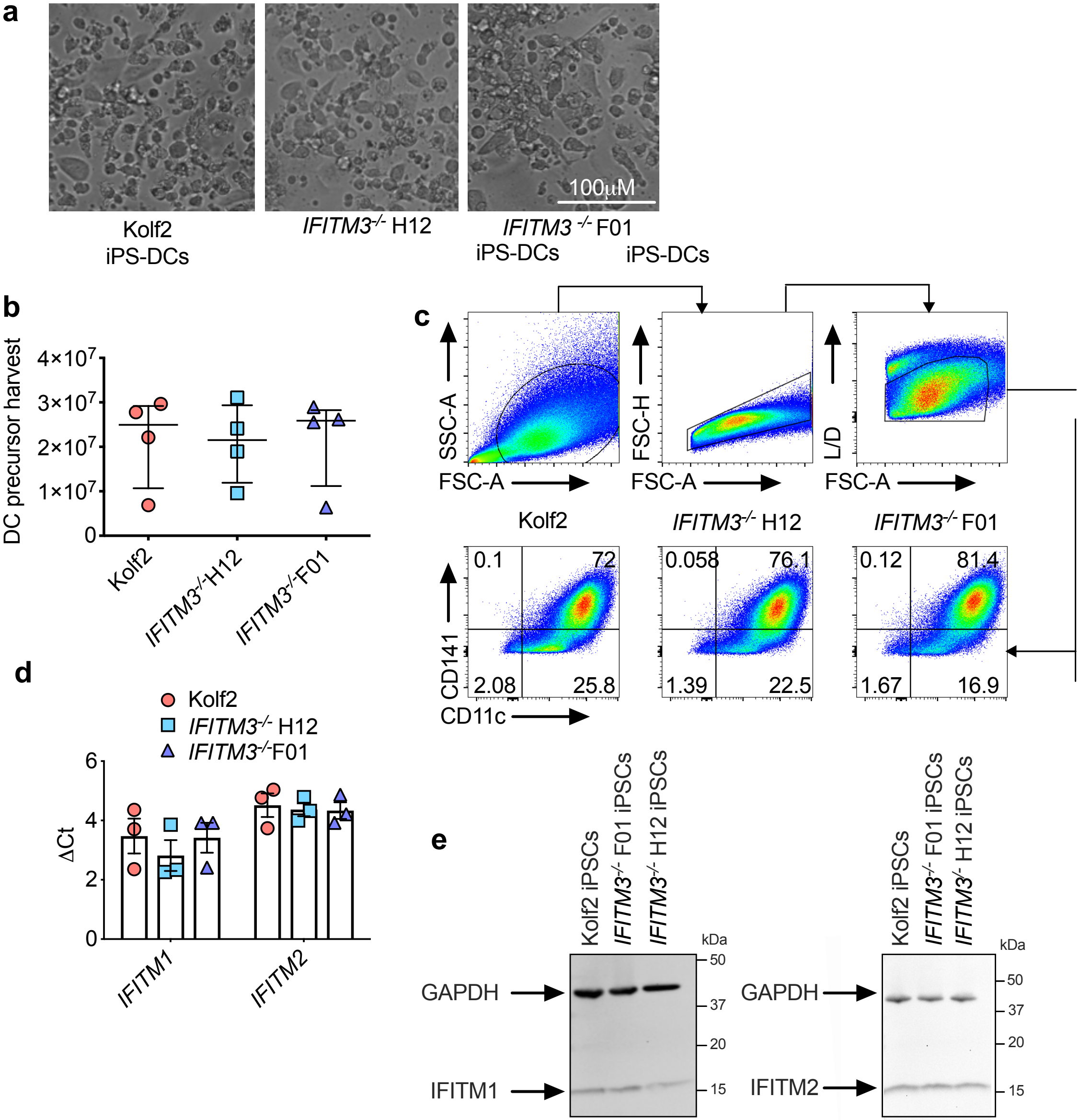

a
100μM
Kolf2 iPS-DCs
IFITM3-/- H12 iPS-DCs
IFITM3 -/- F01 iPS-DCs
b
DC precursor harvest
4×10^7
3×10^7
2×10^7
1×10^7
0
Kolf2
IFITM3-/-H12
IFITM3-/-F01
c
SSC-A
FSC-A
FSC-H
FSC-A
L/D
FSC-A
Kolf2
IFITM3-/- H12
IFITM3-/- F01
0.1
72
2.08
25.8
0.058
76.1
1.39
22.5
0.12
81.4
1.67
16.9
CD141
CD11c
d
Kolf2
IFITM3-/- H12
IFITM3-/-F01
ΔCt
6
4
2
0
IFITM1
IFITM2
e
Kolf2 iPSCs
IFITM3-/- F01 iPSCs
IFITM3-/- H12 iPSCs
kDa
50
37
20
15
GAPDH
IFITM1
IFITM2

**Supplementary Figure 2. IFITM3-deficiency does not affect DC differentiation and phenotype.** CRISPR/Cas9 was used to generate biallelic mutations in IFITM3 in Kolf2 iPSCs. iPSCs were differentiated into dendritic cells using defined concentrations of growth factors to generate embryoid bodies (EBs), GM-CSF and IL-4 to generate immature DCs from EBs. (**a**) Morphology of iPS-DCs in culture (100X). (**b**) Total cell numbers of DC precursors harvested from DC differentiation plates. Data shown as individual technical replicates + mean and are from 4 independent differentiations per iPSC line. (**c**) Surface expression of DC markers CD11c and CD141 was examined by flow cytometry. Representative plots presented from one experiment, with experiments performed at least three times. (**d**) Gene expression of *IFITM1* and *IFITM2* by iPS-DCs relative to *GAPDH* was quantified using TaqMan gene expression assays. Data presented shows mean Ct values +/- SEM from 3 biologically independent cell cultures, with assays repeated in triplicate. (**e**) IFITM1 and IFITM2 expression in iPSCs was assayed by Western blot. Data represents 2 separate experiments. Source data are provided as a Source Data file.

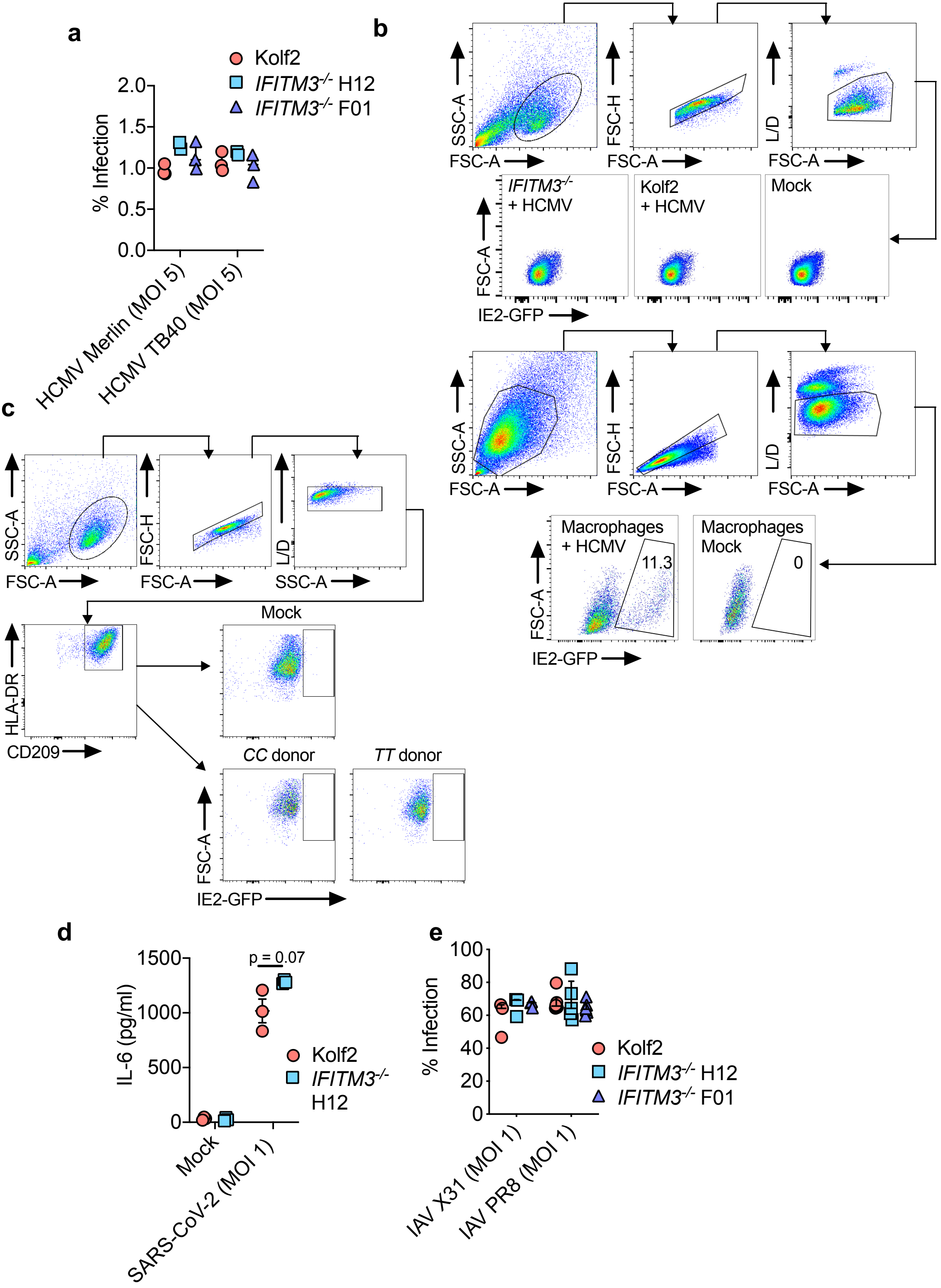
a
Kolf2
IFITM3-/- H12
IFITM3-/- F01
% Infection
2.0
1.5
1.0
0.5
0.0
HCMV Merlin (MOI 5)
HCMV TB40 (MOI 5)
b
SSC-A
FSC-A
FSC-H
L/D
IFITM3-/- + HCMV
Kolf2 + HCMV
Mock
IE2-GFP
Macrophages + HCMV
Macrophages Mock
11.3
0
c
SSC-A
FSC-A
FSC-H
L/D
SSC-A
HLA-DR
CD209
Mock
CC donor
TT donor
FSC-A
IE2-GFP
d
IL-6 (pg/ml)
1500
1000
500
0
p = 0.07
Kolf2
IFITM3-/- H12
Mock
SARS-CoV-2 (MOI 1)
e
% Infection
100
80
60
40
20
0
Kolf2
IFITM3-/- H12
IFITM3-/- F01
IAV X31 (MOI 1)
IAV PR8 (MOI 1)

**Supplementary Figure 3. IFITM3-deficiency in iPS-DCs does not alter infectivity by IAV or HCMV**. (**a&b**) *IFITM3*$^{-/-}$ and Kolf2 iPS-DCs were non-permissive to HCMV using both merlin and TB40, as assayed by expression of IE2-GFP, with representative FACS plots from these assays, and a permissive cell line (macrophages), shown in (**b**). (**c)** Monocyte-derived DCs were non-permissive to HCMV, as assayed by expression of IE2-GFP, with representative FACS plots shown for *CC* or *TT* donors. (**d**) iPS-DCs were infected with SARS-CoV-2 (MOI 1), and IL-6 in supernatants was assayed 6h post infection by ELISA. Individual technical replicates + mean are shown and statistical significance was assessed using Student's *t* test performing paired analysis of SARS-CoV-2 stimulated cells. (**e**) *IFITM3*$^{-/-}$ and Kolf2 iPS-DCs were stimulated with IAV A/X31 (MOI 1) and then stained for IAV NP 24h post-stimulation.

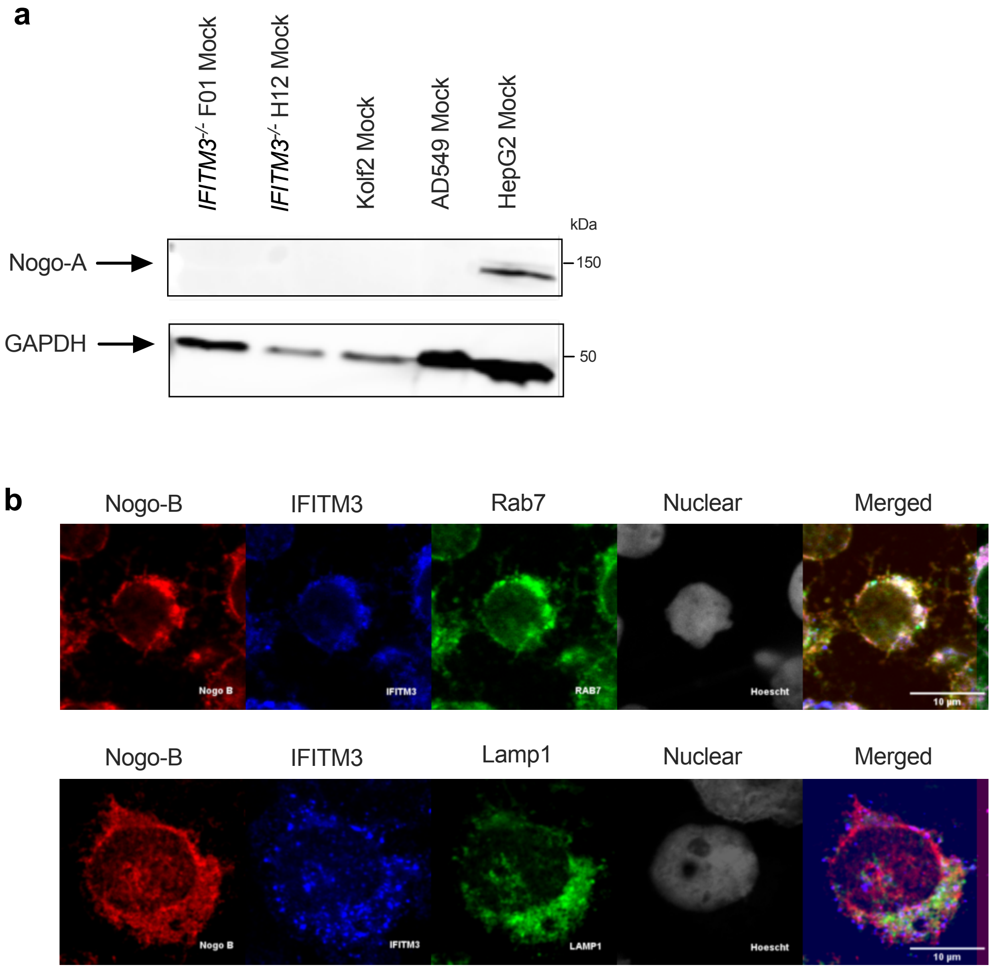


**Supplementary Figure 4. iPS-DCs do not express NOGO-A**. (**a**) Expression of anti-Nogo-A was assayed in iPS-DCs, A549s and HepG2s from whole cell lysates by Western blot. (**b**) Immunoflourescent images of THP1 cells stained with anti-Nogo B, anti-IFITM3 and anti-RAB7 (top) or anti-Lamp1 (bottom), and Hoescht 33342 nuclear stain taken at x60 magnification using a Zeiss LSM 800 confocal microscope. Data represents 2 (**a**) and 3 (**b**) separate experiments.

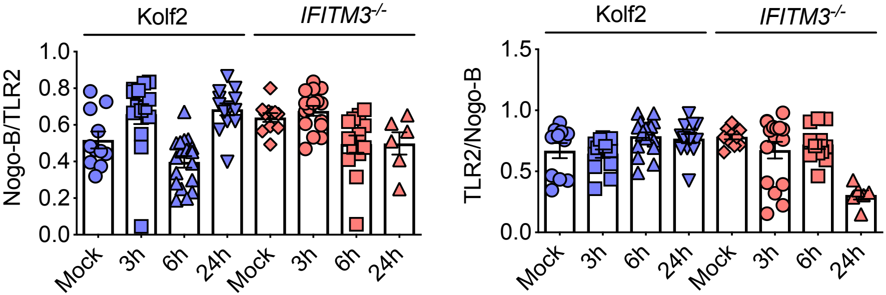


**Supplementary Figure 5. Nogo-B and TLR2 colocalise after HCMV infection.** Immunostaining for TLR2 and NogoB was performed in *IFITM3*$^{-/-}$ and Kolf2 iPS-DCs stimulated for 1h, 3h or 24h with HCMV or mock treated followed by colocalization analysis. Data presented is percentage colocalization for at least 10 cells per condition. Mean +/- SEM are shown from 6-18 different cell cultures. Source data are provided as a Source Data file.

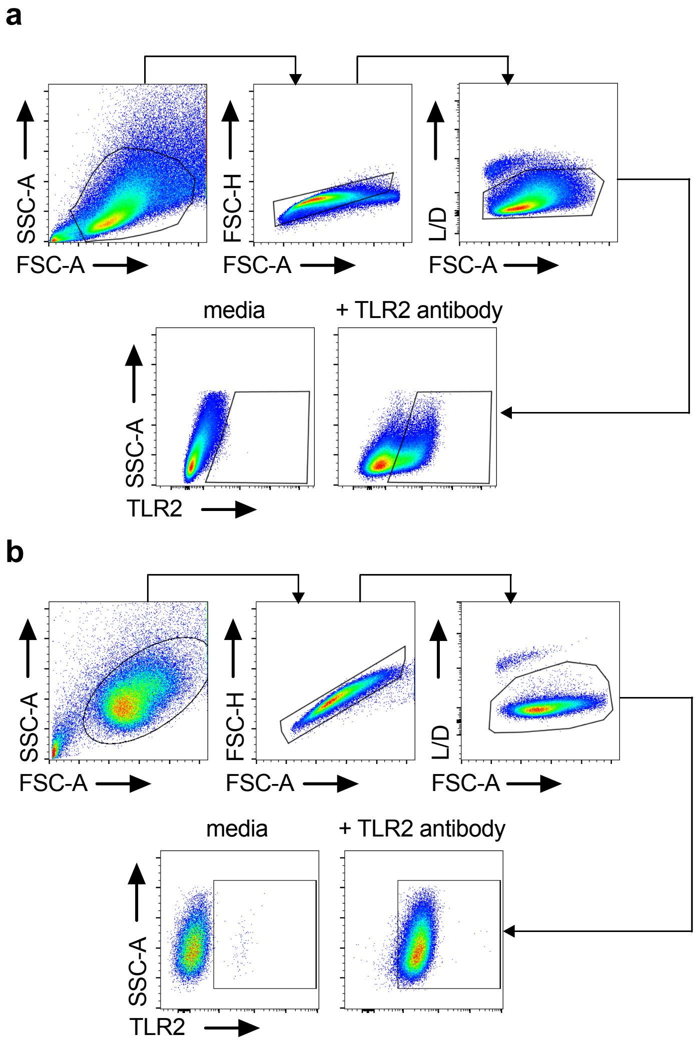


**Supplementary Figure 6. Gating strategy of surface TLR2 assessed by FACS in (a) Kolf2 and *IFITM3*$^{-/-}$ iPS-DCs in Figure 7 b&c and (b) THP-1s in Figure 7 d&e .**

**Supplementary Table 1.** Flow cytometry antibodies.

| Antibody | Company/ Clone | Catalog number | Dilution /amount | Validation |
|---|---|---|---|---|
| anti-Influenza-A FITC | Abcam (431) | ab20921 | 1/50 | https://www.abcam.com/Influenza-A-Virus-Nucleoprotein-antibody-431-FITC-ab20921.html?intFromAbID=81126 |
| TruStain FcX | Biolegend (CD16 (3G8), CD32 (FUN-2) CD64 (10.1)) | 422301 | 1/20 | https://www.biolegend.com/en-gb/products/human-trustain-fcx-fc-receptor-blocking-solution-6462 |
| anti-CD11c FITC | Biolegend (Bu15) | 337213 | 1/50 | https://www.biolegend.com/en-gb/products/fitc-anti-human-cd11c-antibody-6087 |
| anti-CD141 APC | Biolegend (M80) | 344105 | 1/50 | https://www.biolegend.com/en-gb/products/apc-anti-human-cd141-thrombomodulin-antibody-7168 |
| anti-TLR2 PE | Biolegend (TLR2.1) | 309707 | 1/50 | https://www.biolegend.com/en-us/products/pe-anti-human-cd282-tlr2-antibody-1505 |
| anti-TLR2 APC | Biolegend (W15145C) | 392303 | 1/100 | https://www.biolegend.com/en-gb/products/apc-anti-human-cd282-tlr2-antibody-15332 |
| anti-HLA-DR PB | Biolegend (L243) | 307623 | 1/100 | https://www.biolegend.com/en-gb/products/pacific-blue-anti-human-hla-dr-antibody-3335 |
| anti-CD209 DC-SIGN PE-Cy7 | Biolegend (9E9A8) | 330114 | 1/50 | https://www.biolegend.com/en-gb/products/pe-cyanine7-anti-human-cd209-dc-sign-antibody-7059 |

**Supplementary Table 2.** Immunostaining and Imaging antibodies.

| Antibody | Company/ Clone | Catalog number | Dilution/ amount | Validation |
|---|---|---|---|---|
| anti-goat IgG FITC | Abcam | ab6737 | 1/1000 | https://www.abcam.com/rabbit-goat-igg-hl-fitc-ab6737.html |
| anti-TLR2 | Abcam Immunogen CLEIDASD LQSYEPK SLKSIQNV SHLI | ab1655 | 1/100 | https://www.abcam.com/tlr2-antibody-ab1655.html |
| Goat anti-mouse IgG DyLight™ 488 | Biolegend (poly4053) | 405310 | 1/200 | https://www.biolegend.com/en-gb/products/dylight-488-goat-anti-mouse-igg-minimal-x-reactivity-5687 |
| Goat anti-mouse IgG DyLight™ 649 | Biolegend (poly4053) | 405312 | 1/200 | https://www.biolegend.com/en-gb/products/dylight-649-goat-anti-mouse-igg-minimal-x-reactivity-5689 |
| Donkey anti-rabbit IgG DyLight™ 649 | Biolegend (poly4064) | 406406 | 1/200 | https://www.biolegend.com/en-gb/products/dylight-649-donkey-anti-rabbit-igg-minimal-x-reactivity-5695 |
| Donkey anti-rabbit IgG AF488 | Biolegend (poly4064) | 406416 | 1/200 | https://www.biolegend.com/en-gb/products/alexa-fluor-488-donkey-anti-rabbit-igg-minimal-x-reactivity-9380 |
| anti-human CD107a FITC | Biolegend (H4A3) | 328606 | 1/50 | https://www.biolegend.com/en-gb/products/fitc-anti-human-cd107a-lamp-1-antibody-4966 |
| anti-Rab7A | Biolegend (W16034A) | 850401 | 1/100 | https://www.biolegend.com/en-gb/products/purified-anti-rab7a-antibody-14708 |
| Goat anti-rat IgG AF647 | Biolegend (poly4054) | 405416 | 1/200 | https://www.biolegend.com/en-gb/products/alexa-fluor-647-goat-anti-rat-igg-minimal-x-reactivity-9252 |
| anti-Nogo-B | Biotechne Accession # NP_722550 | AF6034 | 1/100 | https://www.rndsystems.com/products/human-mouse-nogo-b-antibody_af6034#product-datasheets |
| anti-sheep IgG Cy5 | Merck Millipore | AP184S | 1/1000 | https://www.merckmillipore.com/GB/en/product/Donkey-Anti-Sheep-IgG-Antibody-Cy5-conjugate-Species-Adsorbed,MM_NF-AP184S |

| | | | | |
|---|---|---|---|---|
| anti-TLR2 | Novus biologicals | NB100-56720 | 1/200 | https://www.novusbio.com/products/tlr2-antibody_nb100-56720#datasheet |
| anti-Sheep IgG (H+L) AF594 | Thermo-Fisher | A-11016 | 1/200 | https://www.thermofisher.com/antibody/product/Donkey-anti-Sheep-IgG-H-L-Cross-Adsorbed-Secondary-Antibody-Polyclonal/A-11016 |
| anti-IFITM3 | In house | n/a | 1/200 | Wellington, D. *et al.* IFITM3-specific antibody reveals IFN preferences and slow IFN induction of the antiviral factor IFITM3 in humans. *Eur J Immunol* **51**, 742-745 (2021). |

**Supplementary Table 3.** Protein preparation and western blotting antibodies.

| Antibody | Company/ Clone | Catalog number | Dilution/ amount | Validation |
|---|---|---|---|---|
| anti-fragilis | Abcam | ab15592 | 1µg/ml | https://www.abcam.com/fragilis-antibody-ab15592.html |
| Veriblot IP detection reagent | Abcam | ab131366 | 1/200 | https://www.abcam.com/veriblot-for-ip-detection-reagent-hrp-ab131366.html |
| anti-Nogo-A | Abcam Accession # NP_065393 | ab62024 | 1µg/ml | https://www.abcam.com/nogo-a-antibody-ab62024.html |
| anti-sheep IgG (H+L)-HRP | Biorad | 1721017 | 1/3000 | https://www.bio-rad.com/en-uk/sku/1721017-rabbit-anti-sheep-igg-hl-hrp-conjugate?ID=1721017 |
| anti-rabbit IgG (H+L)-HRP | Biorad | STAR124P | 1/3000 | https://www.bio-rad-antibodies.com/polyclonal/rabbit-lapine-igg-antibody-star124.html?f=hrp&_ga=2.175517666.1766291682.1655199188-1864392910.1655199187 |
| anti-Nogo-B | Biotechne Accession # NP_722550 | AF6034 | 0.2µg/ml | https://www.rndsystems.com/products/human-mouse-nogo-b-antibody_af6034#product-datasheets |
| anti-sheep (H+L) AF680 | Invitrogen | A-21102 | 0.2µg/ml | https://www.thermofisher.com/antibody/product/Donkey-anti-Sheep-IgG-H-L-Cross-Adsorbed-Secondary-Antibody-Polyclonal/A-21102 |
| IRDye 680LT goat anti-mouse | Li-Cor | 926-68020 | 1/5000 | https://www.licor.com/bio/reagents/irdye-680lt-goat-anti-mouse-igg-secondary-antibody |
| IRDye 800LT goat anti-rabbit | Li-Cor | 925-32210 | 1/5000 | https://www.licor.com/bio/reagents/irdye-800cw-goat-anti-rabbit-igg-secondary-antibody |
| anti-GAPDH | Merck Millipore | MAB374 | 1µg/ml | https://www.merckmillipore.com/GB/en/product/Anti-Glyceraldehyde-3-Phosphate-Dehydrogenase-Antibody-clone-6C5,MM_NF-MAB374 |
| anti-Actin | Merck Millipore | A2066 | 1µg/ml | https://www.sigmaaldrich.com/GB/en/product/sigma/a2066?gclid=Cj0KCQjwwJuVBhCAARIsAOPwGASaWVYIB9SqfPapSGQREbytgqIJeJH3003yBBA-k3DpD8xX6kF-S1EaAp3MEALw_wcB |

| | | | | |
|---|---|---|---|---|
| anti-IFITM1 | Proteintech (5B5E2) | 60074-1-lg | 3μg/ml | https://www.ptglab.com/products/IFITM1-Antibody-60074-1-Ig.htm |
| anti-IFITM2 | Proteintech | 12769-1-AP | 3μg/ml | https://www.ptglab.com/products/IFITM2-Antibody-12769-1-AP.htm |

**Supplementary Table 4.** Proteomic pulldown antibodies.

| Antibody | Company/ Clone | Catalog number | Dilution/ amount | Validation |
|---|---|---|---|---|
| anti-fragilis | Abcam | ab15592 | 1µg/ml | https://www.abcam.com/fragilis-antibody-ab15592.html |
| anti-rabbit IgG | Abcam | ab37415 | 1µg/ml | https://www.abcam.com/rabbit-igg-polyclonal-isotype-control-ab37415.html |

**Supplementary Table 5**. Neutralization assay antibodies.

| Antibody | Company/ Clone | Catalog number | Dilution/ amount | Validation |
|---|---|---|---|---|
| Cytotect | Biotest | 626010010 | 500µg/ml | https://www.biotest.com/gb/en/products/clinical_immunology/cytotect_cp-biotest/product_profile.cfm |
| Anakinra/ KINERET | Cardiff & Vale NHS Pharmacy | n/a | 500ng/ml | https://www.kineretrx.com |
| anti-TLR2 | Invivogen | pab-hstlr2 | 200µg/ml | https://www.invivogen.com/anti-tlr2 |

**Supplementary Table 6**. gRNA sequence for generation of *IFITM3*$^{-/-}$ iPSCs and primer sequences for validation of *IFITM3*$^{-/-}$ iPSCs, and *IFITIM3* genotyping primers.

| ***IFITM3*$^{-/-}$iPS DC generation** | **Sequence** |
|---|---|
| gRNA sequence | TGGGGCCATACGCACCTTCA CGG |
| External Primer (F) | TGTGAGTTCCCTTCTCACTTT |
| External Primer (R) | CACTGTCCAAACCTTCTTCTC |
| Internal Primer (F) | AGTCACAGGGACACACAAGTC |
| Internal Primer (R) | CAACACCCTCTTCATGAACCC |
| ***IFITM3* genotyping primers** | |
| Forward | GGCAGAGGTGAGGGCTTT |
| Reverse | GTCCCTTACGAGTCTCCCAC |

**Supplemental Table 7**. Sequence information of *IFITM3*$^{-/-}$ iPSC clones. Underlined sequences are CD225 domain, and alternative methionine start codon in some protein isoforms.

| **IFITM3** | **Sequence** |
|---|---|
| IFITM3_WT sequence | CCTCTGAGCATTCCCTGGGGCCATACGCACCTTCACGGAGTAGGCGA |
| IFITM3_F01 MUT sequence | CCTCTGAGCATTCCCTGGGGCCATACGCACCTTTCACGGAGTAGGCGA |
| IFITM3_H12 First Allele MUT sequence (Insertion of T) | CCTCTGAGCATTCCCTGGGGCCATACGCACCTTTCACGGAGTAGGCGA |
| IFITM3_H12 Second Allele MUT sequence (Insertion of T) | CCTCTGAGCATTCCCTGGGGCCATACGCACCTCTTCACGGAGTAGGCG |
| WT Protein | MNHTVQTFFSPVNSGQPPNYEMLKEEHEVAVLGAPHNPAPPTSTVIHIRSETSVPDHVVWSLFNTLFMNPCCLGFIAFAYSVKSRDRKMVGDVTGAQAYASTAKCLNIWALILGILMTILLIVIPVLIFQAYG* |
| MUT Protein (T Insertion) | MNHTVQTFFSPVNSGQPPNYEMLKEEHEVAVLGAPHNPAPPTSTVIHIRSETSVPDHVVWSLFNTLFMNPCCLGFIAFAYSVKV* |
| MUT Protein (CT Insertion) | MNHTVQTFFSPVNSGQPPNYEMLKEEHEVAVLGAPHNPAPPTSTVIHIRSETSVPDHVVWSLFNTLFMNPCCLGFIAFAYSVKSLGTGRWLAT* |
