# Supplementary material for: IFITM3 restricts virus-induced inflammatory cytokine production by limiting Nogo-B mediated TLR responses
Source: Nat Commun. 2022 Sep 8;13:5294. doi: 10.1038/s41467-022-32587-4 (PMC9454482; doi:10.1038/s41467-022-32587-4)
Supplement: Supplementary file 3 — Reporting Summary [file 41467_2022_32587_MOESM3_ESM.pdf]

nature portfolio

Corresponding author(s): Ian Humphreys

Last updated by author(s): 20/07/22

Reporting Summary

Nature Portfolio wishes to improve the reproducibility of the work that we publish. This form provides structure for consistency and transparency in reporting. For further information on Nature Portfolio policies, see our Editorial Policies and the Editorial Policy Checklist.

Statistics

For all statistical analyses, confirm that the following items are present in the figure legend, table legend, main text, or Methods section.

n/a	Confirmed	
☐	☒	The exact sample size (*n*) for each experimental group/condition, given as a discrete number and unit of measurement
☐	☒	A statement on whether measurements were taken from distinct samples or whether the same sample was measured repeatedly
☐	☒	The statistical test(s) used AND whether they are one- or two-sided<br>*Only common tests should be described solely by name; describe more complex techniques in the Methods section.*
☒	☐	A description of all covariates tested
☐	☒	A description of any assumptions or corrections, such as tests of normality and adjustment for multiple comparisons
☐	☒	A full description of the statistical parameters including central tendency (e.g. means) or other basic estimates (e.g. regression coefficient) AND variation (e.g. standard deviation) or associated estimates of uncertainty (e.g. confidence intervals)
☐	☒	For null hypothesis testing, the test statistic (e.g. *F*, *t*, *r*) with confidence intervals, effect sizes, degrees of freedom and *P* value noted<br>*Give P values as exact values whenever suitable.*
☒	☐	For Bayesian analysis, information on the choice of priors and Markov chain Monte Carlo settings
☒	☐	For hierarchical and complex designs, identification of the appropriate level for tests and full reporting of outcomes
☒	☐	Estimates of effect sizes (e.g. Cohen's *d*, Pearson's *r*), indicating how they were calculated

Our web collection on statistics for biologists contains articles on many of the points above.

Software and code

Policy information about availability of computer code

Data collection	Equipment:<br>HCMV propagation: Plaques were visualized using a Zeiss Axio Observer Z1 microscope<br>Generation of IFITM3-/- iPSCs: Illumina MiSeq<br>Flow cytometry: Data was acquired using an Attune NxT flow cytometer (v3.2.1)<br>Immunostaining: Images were captured using a Zeiss LSM 800 confocal microscope or an DeltaVision Elite system, with images captured using a CoolSNAP HQ2 camera
Data analysis	Software used:<br>Zen blue (v2.6), ImageJ (v2.1.0/1.53f51), FlowJo (v10.5.3), GraphPad Prism (v8.1.1), MaxQuant (v2.0.1.0), Huygens deconvolution software (v14.10)

For manuscripts utilizing custom algorithms or software that are central to the research but not yet described in published literature, software must be made available to editors and reviewers. We strongly encourage code deposition in a community repository (e.g. GitHub). See the Nature Portfolio guidelines for submitting code & software for further information.

Data

Policy information about availability of data

All manuscripts must include a data availability statement. This statement should provide the following information, where applicable:

- Accession codes, unique identifiers, or web links for publicly available datasets
- A description of any restrictions on data availability
- For clinical datasets or third party data, please ensure that the statement adheres to our policy

The mass spectrometry proteomics data have been deposited to the ProteomeXchange Consortium via the PRIDE partner repository under the dataset identifier PXD035254 (https://www.ebi.ac.uk/pride/). All data are included in the Supplemental Information or available from the authors upon reasonable requests, as are unique reagents used in this Article. Source data are provided with this paper. The raw numbers for charts and graphs are available in the Source Data file whenever possible.

Field-specific reporting

Please select the one below that is the best fit for your research. If you are not sure, read the appropriate sections before making your selection.

☒ Life sciences ☐ Behavioural & social sciences ☐ Ecological, evolutionary & environmental sciences

For a reference copy of the document with all sections, see nature.com/documents/nr-reporting-summary-flat.pdf

Life sciences study design

All studies must disclose on these points even when the disclosure is negative.

Sample size	Power analyses was performed by the Systems Immunity University Research Institute statistician. Data used in these studies was obtained from a similar study by our group (Stacey et al, J Clin Invest, 2017) where the model for IFITM3 regulated pathogenesis was first described. For in vitro assays with virus-infected myeloid cells including iPS-derived cells, we used group numbers based on a previous publication that used these cell systems (Forbester et al, J Vir, 2020).
Data exclusions	No data was excluded
Replication	Experiments were repeated multiple times, as described in the Figure Legends
Randomization	Mice were randomly assigned into groups by an individual from the laboratory who was not directly involved in the study. For in vitro studies (e.g., ELISA, Western blots), conditions/treatment were sometimes performed in different well placements. For human studies, blinding was not possible (e.g., for analysis of IFITM3 genotypes. All studies were also performed using multiple batches of cells, reagents/inhibitors etc to control for covariates.
Blinding	Blinding was performed wherever possible. Number systems were used rather than mouse/sample IDs and assays such as ELISAs and plaque assays were performed by individuals unfamiliar with the experiments, hypotheses etc. For all in vitro experiments, blinding was performed for ELISAs by individuals unaware of the experimental setup performing ELISAs where possible. Microscopy experiments were performed by individuals unaware of the experimental design. Expertise in Western blots was more limited in the lab and was not possible. However, all data were quantified independently to minimize bias.

Reporting for specific materials, systems and methods

We require information from authors about some types of materials, experimental systems and methods used in many studies. Here, indicate whether each material, system or method listed is relevant to your study. If you are not sure if a list item applies to your research, read the appropriate section before selecting a response.

Materials & experimental systems

n/a	Involved in the study	
☐	☒	Antibodies
☐	☒	Eukaryotic cell lines
☒	☐	Palaeontology and archaeology
☐	☒	Animals and other organisms
☐	☒	Human research participants
☒	☐	Clinical data
☒	☐	Dual use research of concern

Methods

n/a	Involved in the study	
☒	☐	ChIP-seq
☐	☒	Flow cytometry
☒	☐	MRI-based neuroimaging

Antibodies

Antibodies used

Flow cytometry:
anti-Influenza-A FITC (Abcam (431) ab20921, 1/50), TruStain FcX (Biolegend (CD16 (3G8), CD32 (FUN-2) CD64 (10.1)) 422301, 1/20), anti-CD11c FITC (Biolegend (Bu15) 337213, 1/50), anti-CD141 APC (Biolegend (M80) 344105, 1/50), anti-TLR2-PE (Biolegend (TLR2.1) 309707, 1/50), anti-TLR2-APC (Biolegend (W15145C), 392303, 1/100), anti-HLA-DR PB (Biolegend (L243) 307623, 1/100), anti-CD209 DC-SIGN PE-Cy7 (Biolegend (9E9A8) 330114, 1/50).

Immunostaining and Imaging:
anti-goat IgG FITC (Abcam, ab6737, 1/1000), anti-TLR2 (Abcam, Immunogen CLEIDASDLQSYEPKSLKSIQNVSHLI, ab1655, 1/100), Goat anti-mouse IgG DyLight™ 488 (Biolegend (poly4053), 405310, 1/200), Goat anti-mouse IgG DyLight™ 649 (Biolegend (poly4053), 405312, 1/200), Donkey anti-rabbit IgG DyLight™ 649 (Biolegend (poly4064), 406406, 1/200), Donkey anti-rabbit IgG AF488 (Biolegend (poly4064), 406416, 1/200), anti-human CD107a FITC (Biolegend (H4A3), 328606, 1/50), anti-Rab7A (Biolegend (W16034A), 850401, 1/100), Goat anti-rat IgG AF647 (Biolegend (poly4054), 405416, 1/200), anti-Nogo-B (Biotechne (Accession # NP_722550), AF6034, 1/100), anti-sheep IgG Cy5 (Merck Millipore, AP184S, 1/1000), anti-TLR2 (Novus biologicals, NB100-56720, 1/200), anti-Sheep IgG (H+L) AF594 (Thermo-Fisher, A-11016, 1/200), anti-IFITM3 (In house, 1/200).

Protein preparation and western blotting:
anti-fragilis (Abcam, ab15592, 1µg/ml), Veriblot IP detection reagent (Abcam, ab131366, 1/200), anti-Nogo-A (Abcam (Accession # NP_065393), ab62024, 1µg/ml), anti-sheep IgG (H+L)-HRP (Biorad, 1721017, 1/3000), anti-rabbit IgG (H+L)-HRP (Biorad, STAR124P, 1/3000), anti-Nogo-B (Biotechne (Accession # NP_722550), AF6034, 0.2µg/ml), anti-sheep (H+L) AF680 (Invitrogen, A-21102, 0.2µg/ml), IRDye 680LT goat anti-mouse (Li-Cor, 926-68020, 1/5000), IRDye 800LT goat anti-rabbit (Li-Cor, 925-32210, 1/5000), anti-GAPDH (Merck Millipore, MAB374, 1µg/ml), anti-Actin (Merck Millipore, A2066, 1µg/ml), anti-IFITM1 (Proteintech (5B5E2), 60074-1-lg, 3µg/ml), anti-IFITM2 (Proteintech, 12769-1-AP, 3µg/ml),

Protein Pull downs:
anti-fragilis (Abcam, ab15592, 1µg/ml), anti-rabbit IgG (Abcam, ab37415, 1µg/ml)

Neutralization:
Cytotect (Biotest, 626010010, 500ug/ml), Anakinra/KINERET (Cardiff & Vale NHS Pharmacy, 500ng/ml), anti-TLR2 (Invivogen, pab-hstlr2, 200ug/ml)

Validation

Validation was performed for all antibodies used by the source company, i.e.

Flow cytometry:
https://www.abcam.com/Influenza-A-Virus-Nucleoprotein-antibody-431-FITC-ab20921.html?intFromAbID=81126
https://www.biolegend.com/en-gb/products/human-trustain-fcx-fc-receptor-blocking-solution-6462
https://www.biolegend.com/en-gb/products/fitc-anti-human-cd11c-antibody-6087
https://www.biolegend.com/en-gb/products/apc-anti-human-cd141-thrombomodulin-antibody-7168
https://www.biolegend.com/en-us/products/pe-anti-human-cd282-tlr2-antibody-1505
https://www.biolegend.com/en-gb/products/pacific-blue-anti-human-hla-dr-antibody-3335
https://www.biolegend.com/en-gb/products/pe-cyanine7-anti-human-cd209-dc-sign-antibody-7059

Immunostaining and Imaging:
https://www.abcam.com/rabbit-goat-igg-hl-fitc-ab6737.html
https://www.abcam.com/tlr2-antibody-ab1655.html
https://www.biolegend.com/en-gb/products/dylight-488-goat-anti-mouse-igg-minimal-x-reactivity-5687
https://www.biolegend.com/en-gb/products/dylight-649-goat-anti-mouse-igg-minimal-x-reactivity-5689
https://www.biolegend.com/en-gb/products/dylight-649-donkey-anti-rabbit-igg-minimal-x-reactivity-5695
https://www.biolegend.com/en-gb/products/alexa-fluor-488-donkey-anti-rabbit-igg-minimal-x-reactivity-9380
https://www.biolegend.com/en-gb/products/fitc-anti-human-cd107a-lamp-1-antibody-4966
https://www.biolegend.com/en-gb/products/purified-anti-rab7a-antibody-14708
https://www.biolegend.com/en-gb/products/alexa-fluor-647-goat-anti-rat-igg-minimal-x-reactivity-9252
https://www.rndsystems.com/products/human-mouse-nogo-b-antibody_af6034#product-datasheets
https://www.merckmillipore.com/GB/en/product/Donkey-Anti-Sheep-IgG-Antibody-Cy5-conjugate-Species-Adsorbed,MM_NF-AP184S
https://www.novusbio.com/products/tlr2-antibody_nb100-56720#datasheet
https://www.thermofisher.com/antibody/product/Donkey-anti-Sheep-IgG-H-L-Cross-Adsorbed-Secondary-Antibody-Polyclonal/A-11016

In house anti-IFITM3 - Wellington, D. et al. IFITM3-specific antibody reveals IFN preferences and slow IFN induction of the antiviral factor IFITM3 in humans. Eur J Immunol 51, 742-745 (2021).

Protein preparation and western blotting:
https://www.abcam.com/fragilis-antibody-ab15592.html
https://www.abcam.com/veriblot-for-ip-detection-reagent-hrp-ab131366.html
https://www.abcam.com/nogo-a-antibody-ab62024.html
https://www.bio-rad.com/en-uk/sku/1721017-rabbit-anti-sheep-igg-hl-hrp-conjugate?ID=1721017
https://www.bio-rad-antibodies.com/polyclonal/rabbit-lapine-igg-antibody-star124.html?

f=hrp&_ga=2.175517666.1766291682.1655199188-1864392910.1655199187
https://www.rndsystems.com/products/human-mouse-nogo-b-antibody_af6034#product-datasheets
https://www.thermofisher.com/antibody/product/Donkey-anti-Sheep-IgG-H-L-Cross-Adsorbed-Secondary-Antibody-Polyclonal/A-21102
https://www.licor.com/bio/reagents/irdye-680lt-goat-anti-mouse-igg-secondary-antibody
https://www.licor.com/bio/reagents/irdye-800cw-goat-anti-rabbit-igg-secondary-antibody
https://www.merckmillipore.com/GB/en/product/Anti-Glyceraldehyde-3-Phosphate-Dehydrogenase-Antibody-clone-6C5,MM_NF-MAB374
https://www.sigmaaldrich.com/GB/en/product/sigma/a2066?gclid=Cj0KCQjwwJuVBhCAARIsAOPwGASaWVYIB9SqfPapSGQREbytgqIJeJH3003yBBA-k3DpD8xX6kF-S1EaAp3MEALw_wcB
https://www.ptglab.com/products/IFITM1-Antibody-60074-1-Ig.htm
https://www.ptglab.com/products/IFITM2-Antibody-12769-1-AP.htm

Protein Pull downs:
https://www.abcam.com/fragilis-antibody-ab15592.html
https://www.abcam.com/rabbit-igg-polyclonal-isotype-control-ab37415.html
IgG controls were used as were Ifitm3- and Nogo-B deficient mice, as described in the paper.

Neutralization:
https://www.biotest.com/gb/en/products/clinical_immunology/cytotect_cp-biotest/product_profile.cfm
also see (J Clin Invest. 2021 Feb 15;131(4):e139296.)
https://www.kineretrx.com
https://www.invivogen.com/anti-tlr2

Eukaryotic cell lines

Policy information about cell lines

Cell line source(s)	THP1 (ATCC), KOLF2 iPS cells (HipSci), IFITM3-/- iPS cells (HipSci)
Authentication	THP1 cells came from and were authenticated by ATCC, KOLF2 authenticated by the HipSci project. IFITM3 iPSC knockout lines were generated, characterized and banked by the Gene editing core facility at the Wellcome Sanger Institute.
Mycoplasma contamination	Cell lines were tested for mycoplasma (and confirmed negative) prior to experiments being performed.
Commonly misidentified lines (See ICLAC register)	No commonly misidentified cell lines were used.

Animals and other organisms

Policy information about studies involving animals; ARRIVE guidelines recommended for reporting animal research

Laboratory animals	Ifitm3-deficient (Ifitm3-/-) and wt control mice male, and Nogo-A/B and WT controls were used and crossed together to make double kos (Ifitm3-/-Nogo-A/B-/-), and Ifitm3 and MyD88-/- mice were crossed to make Ifitm3-/-MyD88-/- mice. Male and female mice have been used in in vitro and in vivo experiments, with all key findings repeated in both genders. Tlr3-/-, Tlr7-/-and Tlr9-/- mice were also used (female only). Finally we used zDC-DTR mice for chimera experiments (female only).
Wild animals	No wild animals were used.
Field-collected samples	No field collection samples were collected.
Ethics oversight	All animal studies were performed at Cardiff University (Heath Park research support facility) under UK Home Office Project License number P7867DADD, as approved by the UK Home Office, London, United Kingdom.

Note that full information on the approval of the study protocol must also be provided in the manuscript.

Human research participants

Policy information about studies involving human research participants

Population characteristics	There was no population characteristics relevant
Recruitment	iPS Cell work: Unknown. But bias is irrelevant as cells were used to make gene-knockouts therefore experiments were internally controlled for any genetic variables. Also we repeated findings using mice cells in parallel.<br><br>For analysis of CC/TT individuals, all volunteers were provided with full details of the project and provided written informed consent and were not compensated.
Ethics oversight	Written consent was obtained for the use of cell lines for the HipSci project from healthy volunteers. Ethical approval was granted by the National Research Ethics Service (NRES) Research Ethics Committee Yorkshire and The Humber-Leeds West,

under reference number 15/YH/0391.

For analysis of CC/TT individuals, the use of human material was approved by Oxford Tropical Research Ethics Committee (OxTREC) under code OXTREC 1001-13.

Note that full information on the approval of the study protocol must also be provided in the manuscript.

Flow Cytometry

Plots

Confirm that:

- [x] The axis labels state the marker and fluorochrome used (e.g. CD4-FITC).
- [x] The axis scales are clearly visible. Include numbers along axes only for bottom left plot of group (a 'group' is an analysis of identical markers).
- [x] All plots are contour plots with outliers or pseudocolor plots.
- [x] A numerical value for number of cells or percentage (with statistics) is provided.

Methodology

Sample preparation	iPS DCs are non-adherent so cells were gently collected
Instrument	Attune NxT flow cytometer (Thermo-Fisher)
Software	FlowJo
Cell population abundance	These experiments were cultures DCs, so ~75% of cells - see Sup Fig S2
Gating strategy	See S2C. All cells were gated on live cells and then CD141+CD11C+ for the only flow cytometry data in this paper (Figure 7B-C). The other flow work ((Fig 7D-E) was performed with a single cell line (THP1)

- [x] Tick this box to confirm that a figure exemplifying the gating strategy is provided in the Supplementary Information.
